# Supplementary material for: The interactome of intact mitochondria by cross-linking mass spectrometry provides evidence for coexisting respiratory supercomplexes
Source: Mol Cell Proteomics. 2017 Dec 8;17(2):216–32. doi: 10.1074/mcp.RA117.000470 (PMC5795388; doi:10.1074/mcp.RA117.000470)
Supplement: Supplemental Data [file supp_RA117.000470_133922_0_supp_23978_fzffwf.zip › spectra_annotation/mito_DR_spectra_annotation/68-1-8-1-12-1.pdf]

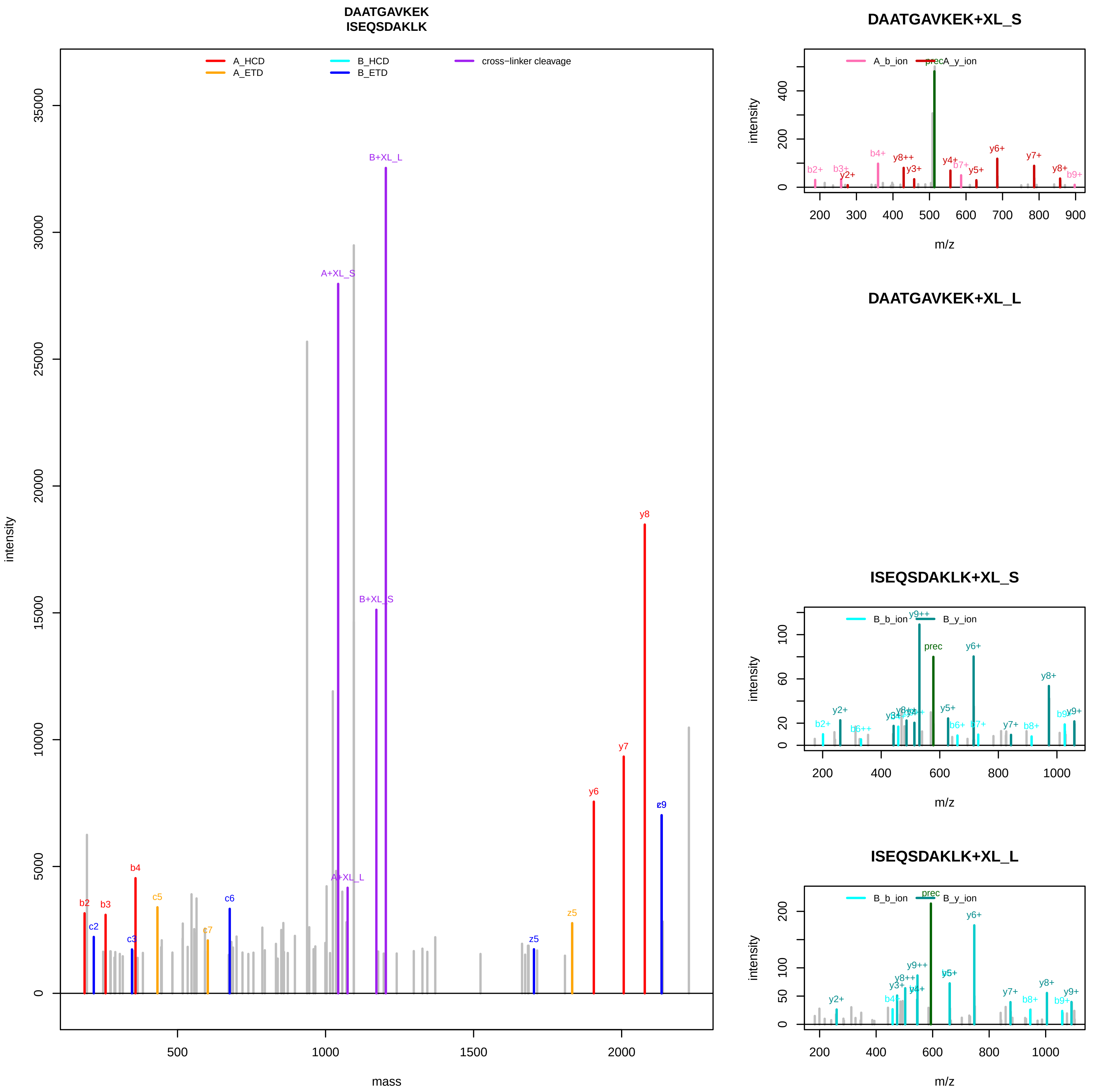
DAATGAVKEK
ISEQSDAKLK
A_HCD
A_ETD
B_HCD
B_ETD
cross−linker cleavage
intensity
mass
b2
b3
b4
c2
c3
c5
c6
c7
z5
z5
y6
y7
y8
z9
A+XL_S
A+XL_L
B+XL_S
B+XL_L
DAATGAVKEK+XL_S
A_b_ion
A_y_ion
prec
b2+
b3+
b4+
b7+
b9+
y2+
y3+
y4+
y5+
y6+
y7+
y8+
y8++
m/z
DAATGAVKEK+XL_L
ISEQSDAKLK+XL_S
B_b_ion
B_y_ion
prec
y9++
y2+
y5+
y6+
y7+
y8+
y9+
b2+
b6+
b6++
b7+
b8+
b9+
m/z
ISEQSDAKLK+XL_L
B_b_ion
B_y_ion
prec
y2+
y3+
y4+
y5+
y6+
y7+
y8+
y8++
y9+
y9++
b4+
b8+
b9+
m/z
